# The Cost-Effectiveness of Tislelizumab Plus Chemotherapy for Locally Advanced or Metastatic Nonsquamous Non-Small Cell Lung Cancer

**DOI:** 10.3389/fphar.2022.935581

**Published:** 2022-07-22

**Authors:** Xia Luo, Zhen Zhou, Xiaohui Zeng, Qiao Liu

**Affiliations:** ^1^ Department of Pharmacy, The Second Xiangya Hospital of Central South University, Changsha, China; ^2^ Menzies Institute for Medical Research, University of Tasmania, Hobart, TAS, Australia; ^3^ Department of Nuclear Medicine/PET Image Center, The Second Xiangya Hospital of Central South University, Changsha, China

**Keywords:** cost-effectiveness, NSqNSCLC, tislelizumab, PD-L1 expression, smoking status, China

## Abstract

**Objective:** To investigate the cost-effectiveness of adding Chinese-developed anti-PD-1 antibody tislelizumab to first-line pemetrexed-platinum chemotherapy in (1) a study population of patients with locally advanced or metastatic nonsquamous non-small cell lung cancer (nsqNSCLC) and without known sensitizing EGFR mutations or ALK rearrangements and (2) its subgroups from the perspective of Chinese healthcare system.

**Material and Methods:** Separate Markov models were constructed for the entire study population and its subgroups; 10,000 patients with locally advanced or metastatic nsqNSCLC and without driver gene mutations were simulated in the first-line tislelizumab plus pemetrexed-platinum (TPP) arm and first-line pemetrexed-platinum (PP) arm, respectively. Transition probabilities were extracted from the RATIONALE 304 trial. Public health state utilities and costs were obtained from published literature, public national databases, and local general hospitals. The main outputs were incremental cost-effectiveness ratios (ICERs). The ICERs were compared to a willingness-to-pay threshold of $35,663 per quality-adjusted life-years (QALYs) to determine the cost-effective treatment. Sensitivity analyses were employed to assess the uncertainty in the model.

**Results:** For the entire patient population, first-line TPP versus PP use increased the effectiveness by 0.99 QALYs and healthcare costs by $28,749, resulting in an ICER of $28,749/QALY that was lower than the prespecified WTP threshold. For patient subgroups, first-line TPP conferred the greatest survival benefit in patients with PD-L1 expression ≥50%, followed by patients with liver metastasis and those who are current or former smokers. Overall, the ICERs for the first-line TPP versus PP ranged from $27,018/QALYs to $33,074/QALYs, which were consistently below the WTP threshold.

**Conclusion:** For Chinese patients with locally advanced or metastatic nsqNSCLC who had no known sensitizing EGFR mutations or ALK rearrangements, adding the Chinese-developed anti-PD-1 antibody tislelizumab to the first-line pemetrexed-platinum chemotherapy was cost-effective regardless of their baseline characteristics.

## Introduction

Lung cancer has consistently been the most fatal cancer in China, with approximately 816,000 new cases and 715,000 deaths recorded in 2020 (Cao et al., 2021; Sung et al., 2021). More than 50% of primary lung cancers are locally advanced or metastatic non-small cell lung cancers (NSCLCs) (Gridelli et al., 2015), of which nearly three-quarters (roughly 300,000 cases) are nonsquamous NSCLCs (nsqNSCLCs) (Chen et al., 2016). A combination therapy of programmed cell death protein 1/programmed cell death-ligand 1 (PD-1/PD-L1) inhibitors with platinum-based chemotherapy is used as a standard-of-care for untreated patients with locally advanced or metastatic nsqNSCLC and without sensitizing epidermal growth factor receptor (EGFR) mutations or anaplastic lymphoma kinase (ALK) rearrangements (Guidelines Working Committee of Chinese Society of Clinical Oncology, 2021; National Comprehensive Cancer Network, 2021). However, the treatment benefit of immunochemotherapy could vary across patient groups with different demographic, genetic, and clinical characteristics such as PD-L1 expression, gender, and smoking status (Patel and Kurzrock, 2015; Gibney et al., 2016; Conforti et al., 2018; Klein and Morgan, 2020; Mo et al., 2020; Hopkins et al., 2022). A better understanding of the modifying effects of patients’ characteristics on treatment effectiveness is crucial to implementing better tailored therapeutic strategies.

Tislelizumab is a China-developed anti-PD-1 antibody that was specifically engineered to minimize FcɣR macrophage binding to abrogate antibody-dependent phagocytosis. Recently, an open-label phase 3 trial (RATIONALE 304; NCT03663205) studied the efficacy and safety of the combination of tislelizumab with pemetrexed-platinum chemotherapy versus pemetrexed-platinum chemotherapy alone in treatment-naive patients with locally advanced or metastatic nsqNSCLC who had no known sensitizing EGFR mutations or ALK rearrangements (Lu et al., 2021). Compared with chemotherapy alone, first-line tislelizumab plus chemotherapy significantly prolonged progression-free survival (PFS) in the entire study population (median PFS: 9.7 versus 7.6 months; hazard ratio [HR] = 0.645) (Lu et al., 2021). However, unlike previous studies combining an anti-PD-1/L1 inhibitor with platinum chemotherapy that found sustained PFS benefits in almost all subgroups (Gandhi et al., 2018a; Herbst et al., 2020; Yang et al., 2020; Zhou et al., 2021), the RATIONALE 304 trial reported no PFS benefits with first-line tislelizumab plus chemotherapy in several subgroups, such as female patients (HR = 0.946; 95% confidence interval (CI): 0.487–1.840), non-smokers (HR = 1.075; 95% CI: 0.596–1.940) and those with a PD-L1 expression ranged from 1 to 49% (HR = 1.058; 95% CI: 0.507–2.209) (Lu et al., 2021). This led to a suggestion that the addition of tislelizumab to chemotherapy would not improve health outcomes in these subgroups of patients while increasing their financial burden.

Although tislelizumab in combination with chemotherapy is recommended as the preferred first-line therapy for locally advanced or metastatic nsqNSCLC by the latest Chinese Society of Clinical Oncology (CSCO) Guidelines (Guidelines Working Committee of Chinese society of Clinical Oncology, 2021), the varying efficacy of the combination therapy in patients with different characteristics suggests that cost-effectiveness studies targeting different subgroups are needed to guide tailored clinical decisions of treatment regimens. Thus, the aim of this study was to evaluate the cost-effectiveness of first-line tislelizumab plus chemotherapy for the entire patient population with locally advanced or metastatic nsqNSCLC and its subgroups from the perspective of the Chinese healthcare system.

## Materials and Methods

### Overview

Using TreeAge Pro 2022 R1 (https://www.treeage.com/) and R version 4.2.0 (http://www.r-project.org), we designed separate Markov models to investigate the cost-effectiveness of adding tislelizumab plus pemetrexed-platinum chemotherapy (TPP) for the entire cohort of patients with locally advanced or metastatic nsqNSCLC and its subgroups by different baseline characteristics (Lu et al., 2021). Markov cohort analyses were performed to compute the cumulative healthcare costs reported in 2021 US dollars and the cumulative effectiveness reflected by the quality-adjusted life-years (QALYs) for each treatment arm within a given time horizon. The cost-effectiveness of first-line TPP was assessed using the incremental cost-effectiveness ratios (ICERs), with an ICER less than the willingness-to-pay (WTP) threshold of $35,663 per QALY (defined as 3 times China’s per capita GDP in 2021) considered cost-effective (Chinese Pharmaceutical Association, 2020; National Bureau of Statistics of China, 2021). We discounted all costs and effectiveness at 5% annually as per the Chinese Guidelines for Pharmacoeconomic Evaluations (Chinese Pharmaceutical Association, 2020).

This economic evaluation collected existing data to inform the model and therefore, was deemed to be exempt from review by the Chinese ethics review committee. This study followed the Chinese Guidelines for Pharmacoeconomic Evaluation (2020) (Chinese Pharmaceutical Association, 2020).

### Model Construction

**FIGURE 1:**
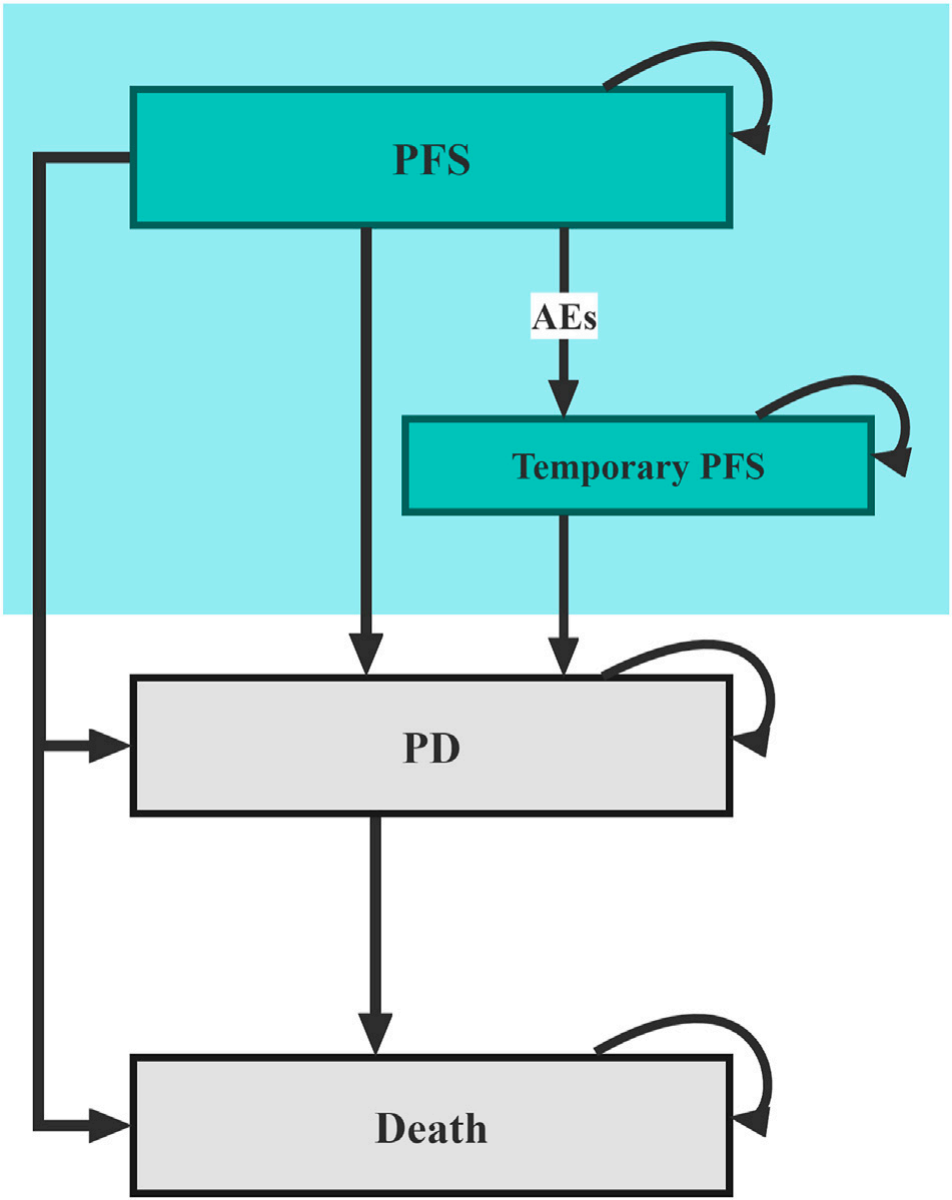
Diagram of the Markov model. PFS, progression-free survival; PD, progressive disease; AE, adverse events.

The model patients mirrored the participants in the RATIONALE 304 trial, who were treatment-naive adult patients (aged 18–75 years) with histologically confirmed locally advanced or metastatic nsqNSCLC and with no known sensitizing EGFR mutations or ALK rearrangements. The structure diagram of the Markov model is shown in Figure 1, in which three main health states composed of PFS, progressive disease (PD), and death were constructed to simulate the disease process of model patients, and a temporary health state of PFS was used to reflect the real-world treatment discontinuation caused by adverse events (AEs). The length of the Markov cycle is set to 21 days according to the dosing interval in the RATIONALE 304 trial (Lu et al., 2021), and the running time of the model is set to 20 years to ensure that more than 99% of the patients reach the expected terminal (health state of death).

Model patients started from the PFS health state and were randomly treated with first-line TPP or the first-line pemetrexed-platinum (PP) (Supplementary Table S1). According to local clinical practice, pemetrexed-treated patients also receive folic acid, vitamin B12, and glucocorticoids as premedication (Supplementary Table S1). During each cycle, patients who had no disease progression but experienced toxicity that was confirmed to be caused by one study drug would transfer to the temporary health state of PFS, in which the study drug was discontinued; patients with disease progression would enter the PD health state and proceed to receive subsequent anticancer therapy. Since the subsequent anticancer therapies data were not published in the RATIONALE 304 trial, we assumed that 50% of the patients with disease progression in both treatment arms received subsequent anticancer therapies. Moreover, according to the national Guidelines for NSCLC, patients would be supplemented with the best supportive care (BSC) when receiving any anticancer treatment and end-of-life care before death (Guidelines Working Committee of Chinese Society of Clinical Oncology, 2021).

### QALYs

In the Markov model, QALYs were calculated as utilities-discounted life-years, which were determined by the transition probabilities and differed by treatment strategies. To construct a parametric survival model for the entire patient population receiving first-line PP, we first reconstructed individual patient-level data based on the Kaplan-Meier (KM) curves reported in the RATIONALE 304 trial (Liu et al., 2021a; Qiao et al., 2021), and then chose the best-fit parametric survival distributions for these data according to the results of goodness-of-fit tests (Supplementary Table S2; Supplementary Figure S1). Finally, we calculated the transition probabilities between the three main health states by using the distribution parameters. For patients receiving first-line TPP, the HRs of OS and PFS for the entire study population, as well as the HR of PFS for its subgroups reported in the RATIONALE 304 trial, were employed for parametric survival modeling. Furthermore, transition probabilities between the two sub PFS health states were derived from the data regarding AEs-related drug discontinuation (Supplementary Table S3).

Chinese-specific health state utilities were captured from a previous study, and the assigned values for PFS health status and PD health status were 0.856 and 0.768, respectively (Shen et al., 2018). The AEs-related disutilities as a result of treatment were also considered in the model and calculated as a frequency-weighted sum, as detailed in Supplementary Table S4 (Nafees et al., 2017). All model inputs for QALYs measurements are summarized in Table 1.

**TABLE 1:** Cost-effectiveness analysis summary.

Treatment	Cost,$	QALYs	Incremental		ICER, $/QALY	Cost-effectiveness probability of first-line TPP (%)
			Cost, $	QALYs		
First-line PP in the entire patient population	37,363	1.79				
First-line TPP in the entire patient population	66,111	2.78	28,749	0.99	29,132	65.59
First-line TPP in different subgroups						
Age <65 years	66,492	2.78	29,129	0.99	29,424	66.49
Age ≥65 years	65,417	2.78	28,054	0.98	28,592	63.83
Female	64,043	2.76	26,680	0.97	27,501	60.23
Male	67,255	2.79	29,892	1.00	30,003	68.15
ECOG performance status:0	64,675	2.77	27,312	0.98	28,007	61.44
ECOG performance status:1	64,675	2.77	27,312	0.98	28,007	65.81
Never smoking	63,447	2.76	26,084	0.97	27,018	64.12
Current or former smoking	68,243	2.80	30,880	1.00	30,738	70.67
ⅢB nsqNSCLC	65,939	2.78	28,576	0.99	28,998	65.24
Ⅳ nsqNSCLC	66,234	2.78	28,871	0.99	29,226	65.14
With liver metastasis	69,993	2.81	32,631	1.02	32,001	73.02
Without liver metastasis	65,401	2.78	28,039	0.98	28,580	63.73
PD-L1 expression <1%	65,185	2.77	27,822	0.98	28,411	62.18
PD-L1 expression ≥1%	67,122	2.79	29,759	1.00	29,902	67.54
PD-L1 expression 1–49%	63,519	2.76	26,156	0.97	27,077	59.04
PD-L1 expression ≥50%	71,538	2.83	34,176	1.03	33,074	74.85
Without ALK rearrangement	66,196	2.78	28,833	0.99	29,197	75.49
Unknown ALK rearrangement	65,894	2.78	28,532	0.99	28,964	74.30

QALY, quality-adjusted life-year; ICER, incremental cost-effectiveness ratio; TPP, tislelizumab plus pemetrexed-platinum chemotherapy; PP, pemetrexed-platinum chemotherapy; ECOG, Eastern Cooperative Oncology Group; NSCLC, non-small cell lung cancer; PD-L1, programmed death-ligand 1; ALK, anaplastic lymphoma kinase.

### Cost

The direct medical costs including drug acquisition, treating AE, and general cancer treatment costs were covered. Drug prices were retrieved from the publicly available national databases (https://www.yaozh.com/index) (China’s health industry data platform, 2021). For drug dosage calculation, we modeled the model patients as having a mean body surface area of 1.72 m^2^ and a mean creatinine clearance rate of 70 ml/min (Liu et al., 2021a; Qiao et al., 2021). The treatment costs of AEs related to each treatment assignment were included in the model using the same algorithm as the disutilities (Supplementary Table S4 **)**.

The general cancer treatment costs commonly include routine follow-ups, subsequent anticancer therapies, BSC, and end-of-life care costs. As per the local guidelines, routine follow-ups included medical history, physical examination, and radiological imaging (Guidelines Working Committee of the Chinese Society of Clinical Oncology, 2021). The costs for routine follow-ups were estimated using data from local general hospitals, and for subsequent anticancer therapies, BSC, and end-of-life care were obtained from previous literature (Qiao et al., 2021).

### Sensitivity Analysis

In this analysis, the validity of our conclusions was determined by the sensitivity analyses. To investigate the influence of the uncertainty of individual parameters on the cost-effectiveness results, we performed separate deterministic sensitivity analyses (DSA) for the HRs, costs, utilities, and other parameters. To test the influence of the uncertainty in multiple parameters on the results, we performed probabilistic sensitivity analyses (PSA) with 10,000 Monte Carlo simulations. The uncertainty of these parameters was reflected by plausible variation ranges and appropriate distributions outlined in Supplementary Table S5 **.**

## Results

### Incremental Cost-Effectiveness Ratios

For the entire study population, the first-line TPP improved the effectiveness by 0.99 QALYs and increased total costs by $28,749 compared with the first-line PP (Table 1). This resulted in an ICER of $28,749/QALY for the first-line TPP versus PP, which was lower than the WTP threshold of $35,663 per QALY used in the analysis.

For subgroup populations, first-line TPP conferred the greatest survival benefit in patients with PD-L1 expression ≥50% (2.83 QALYs), followed by patients with liver metastasis (2.81 QALYs) and those who are current or were former smokers (2.80 QALYs). However, prolonged QALYs were associated with substantially increased healthcare costs (Table 1). Despite this, the ICER between first-line TPP and PP ranged from $27,018/QALYs to $33,074/QALYs, which was consistently below the WTP threshold of $35,663 per QALY.

### Sensitivity Analysis

**FIGURE 2:**
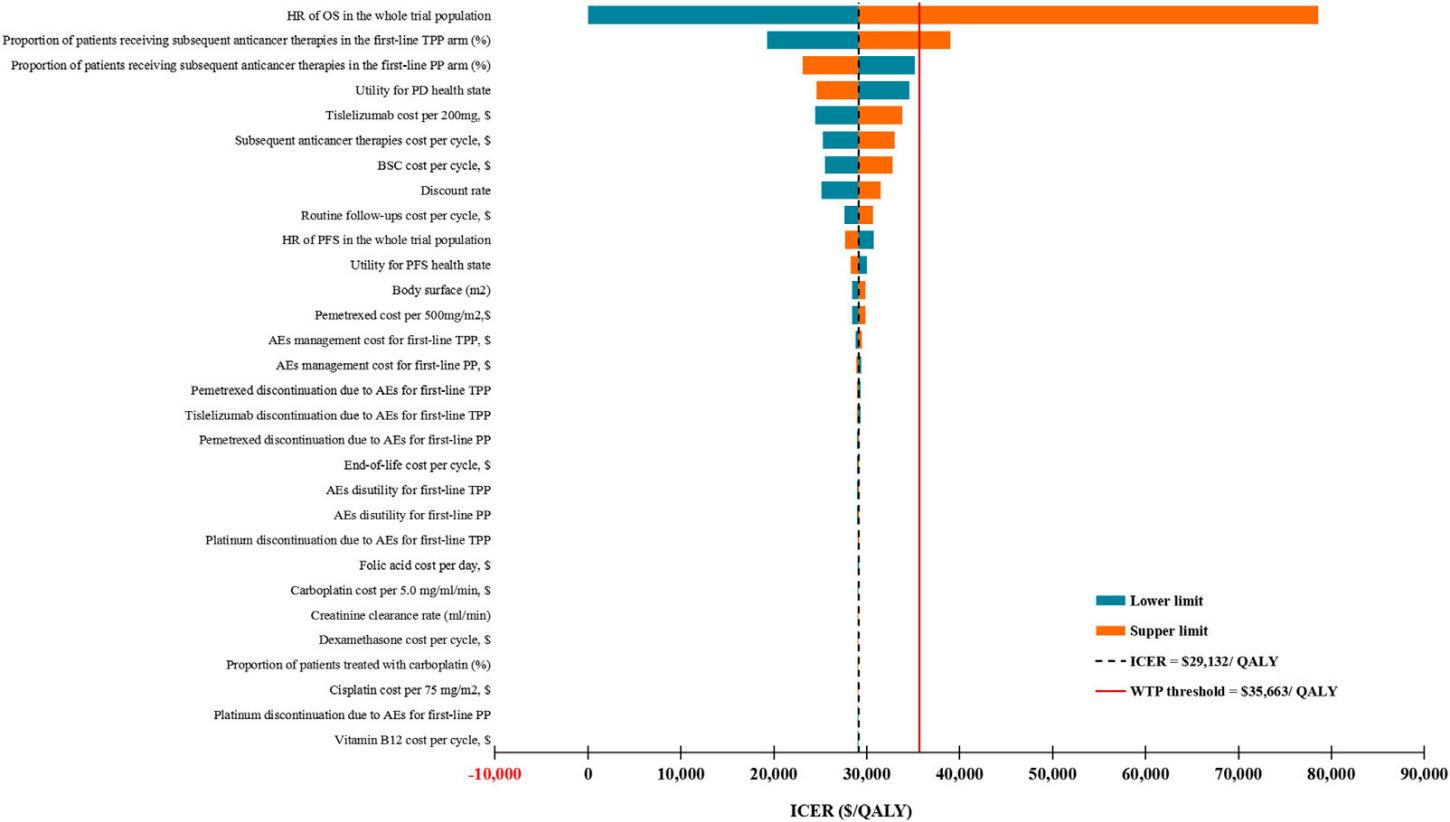
Deterministic sensitivity analysis results for the entire patient population. ICER, incremental cost-effectiveness ratios; QALY, quality-adjusted life-years; WTP, willingness-to-pay; TPP, tislelizumab plus pemetrexed-platinum chemotherapy; PP, pemetrexed-platinum chemotherapy; HR, hazard ratio; OS, overall survival; PFS, progression-free survival; PD, progressive disease; AEs, adverse events; BSC, best supportive care; PD, progressive disease.

For the entire study population, the DSA results showed that the most influential parameters affecting the model robustness were the HR of OS and the proportion of patients receiving subsequent anticancer therapies in the TPP arm **(** Figure 2). We explored the association of these two parameters with the ICER for first-line TPP versus PP, and found that first-line TPP was not cost-effective compared with first-line PP only when the HR of OS exceeded 0.789 or the proportion of patients receiving subsequent anticancer therapies in the TPP arm exceeded 67%. PSA revealed that compared with first-line PP, at the WTP threshold of $35,663 per QALY, the probability that the first-line TPP is cost-effective was 65.59%, and this probability was expected to increase with increasing WTP thresholds (Supplementary Figure S2).

For subgroup populations, the PSA results suggested that first-line TPP had higher probabilities of being cost-effective in subgroups with higher QALYs **(** Table 1 **)**.

## Discussion

The National Bureau of Statistics estimated that China has spent $110 billion on health care in 2020, corresponding to 7% of the national GDP (National Bureau of Statistics of China, 2021). Facing with this tremendous financial burden, the Chinese government and academia have placed a greater emphasis on value-based health care, for which the payments are determined by the quality rather than the quantity of medical resources provided (Liu et al., 2021b). Lung cancer continues to be a major public health problem that threatens millions of people’s lives in China (Cao et al., 2021; Sung et al., 2021). In recent years, PD-1/L1 inhibitor-containing therapies have shown unparalleled clinical efficacy and a favorable safety profile in the management of lung cancer (Steven et al., 2016; Doroshow et al., 2019). Cost-effectiveness analyses for different PD-1/L1-based treatment strategies are needed to inform the treatment regimen that is of the greatest value-for-money (Criss et al., 2019).

This study uniquely estimated the cost-effectiveness of adding tislelizumab (a China-developed anti-PD-1 antibody) to first-line pemetrexed-platinum chemotherapy for patients with locally advanced or metastatic nsqNSCLC from the perspective of the Chinese healthcare system. The cost-effectiveness analyses were repeated in patient subgroups by different baseline characteristics (Lu et al., 2021). We found that the first-line TPP provided approximately an additional 1-year of survival with optimal health for both the entire patient population and its subgroups. Moreover, the ICERs between first-line TPP and PP were consistently below the WTP threshold of $35,663 per QALY, which suggests that the first-line TPP is a preferred strategy regardless of the patient’s baseline characteristics (Table 1). More importantly, this analysis reported the longest QALYs associated with the first-line TPP in the subgroup of patients with PD-L1 expression ≥50%, followed by patients with liver metastasis and those who are current or former smokers (Table 1), which were consistent with the efficacy results of the RATONALE 304 trial (Lu et al., 2021). However, we observed that subgroups with greater survival advantages had higher ICERs for first-line TPP vs. first-line PP. This can be explained by the fact that the longer the time the patient survives, the greater the expenditure they will spend on cancer treatments.

Similar to the KEYNOTE-189 and IMpower130 trials (Gandhi et al., 2018b; West et al., 2019), the RATIONALE 304 trial was also designed to investigate the efficacy and safety of adding a PD-1 inhibitor to conventional chemotherapy in NSCLC patients without sensitizing EGFR or ALK mutations. In particular, the RATIONALE 304 trial recruited patients with locally advanced (ⅢB) nsqNSCLC. The results of this economic evaluation suggested that the ICERs generated by first-line TPP in different disease stages were lower than the WTP threshold of $35,663 per QALY, which supports the use of tislelizumab plus chemotherapy as a first-line treatment option for patients with locally advanced NSCLC who are ineligible for radiotherapy. Furthermore, this analysis also showed favorable ICERs with first-line TPP regardless of ALK rearrangement status, suggesting that the indication of first-line TPP may be generalized to patients with unknown ALK rearrangement. Nevertheless, this conclusion could be validated by further investigation in randomized clinical trials. Although first-line TPP did not show benefits on PFS in the nonsmoker subgroup in the RATIONALE 304 trial (HR = 1.075, 95% confidence interval: 0.596–1.940), the bearish QALY associated with significantly smaller healthcare costs, which enabled the first-line TPP to achieve the lowest ICER in this subgroup.

Sensitivity analyses for assessing parameter uncertainties suggested that only two parameters had the ability to transfer the preferred strategy from first-line TPP to first-line PP, which were HR of OS in the whole study population and the proportion of patients receiving subsequent anticancer therapies in the TPP arm. The present analysis is based on the published data of the RATIONALE 304 trial, in which the OS data and the subsequent anticancer therapy data were immature, underscoring the need for more mature data to verify our model. In addition, our model was not particularly sensitive to anticancer drug costs, which have been proven to play a crucial role in determining the preferred strategy in many previous cost-effectiveness studies (Criss et al., 2019; Wan et al., 2019; Watson et al., 2020; Liu et al., 2021a; Qiao et al., 2021). In the current study, the weakening of the effect of anticancer drug costs on the ICERs may be explained as follows: First, in recent years, the Chinese government has been vigorously supporting the research and development of domestic anticancer drugs (Li et al., 2021) to improve the situation in the past when cancer treatment relied mainly on expensive imported drugs. Second, to promote the reasonable price reduction of novel anticancer drugs, the Chinese government has carried out several rounds of price negotiations with suppliers, leading to the price of many drugs being reduced by more than half (Ministry of Human Resources and Social Security of the People’s Republic of China, 2021). For instance, tislelizumab analyzed in this study was a China-developed anti-PD-1 antibody (Lu et al., 2021). Its listing price in December 2019 was $1657/100mg, which was 40% lower than the price of similar pembrolizumab (China’s health industry data platform, 2021). After a successful negotiation in February 2021, the price was $338/100mg, which was 88% lower than that of pembrolizumab (China’s health industry data platform, 2021).

This study has several limitations. First, it was difficult to identify the potential heterogeneity in the OS across subgroups due to the absence of relevant data; thus, the results of our subgroups analysis should be interpreted with caution. Second, we modeled the proportions of patients receiving subsequent anticancer therapies in both arms as 50%, which may not fully reflect the real-world prevalence; our sensitivity analysis found that the uncertainty in the two parameters may affect our conclusion, emphasizing the necessity of more accurate data. Third, owing to the dearth of quality-of-life data for Chinese patients with locally advanced or metastatic nsqNSCLC, we used the health state utilities from a China-based study (Shen et al., 2018). However, our findings remained robust over wide ranges of these utilities. Fourth, several cost parameters used to inform the model were sourced from published literature, including the costs of subsequent anticancer therapies, BSC, and end-of-life care costs; however, the model seemed insensitive to these model inputs and assumptions. Fifth, we found that the ICERs for TPP use versus PP use in the subgroup of patients with PD-L1 expression <1% were lower than those in the subgroups of patients with PD-L1 expression ≥1% and ≥50%. Because PD-1 inhibitors are typically more effective in improving survival among patients with higher PD-L1 expression (Reck et al., 2016; Gandhi et al., 2018c; Mok et al., 2019; Sezer et al., 2021), our cost-effectiveness evidence should not limit TPP use in this patient group. Sixth, our conclusion may not be generalizable to other countries due to the unique study perspectives and data collected for cost estimation; however, given the highest distribution of new lung cancer cases in China (Cao et al., 2021; Sung et al., 2021), the cost-effectiveness evidence yielded from this study has the potential to help alleviate both national and global disease and financial burdens.

In conclusion, in this study investigating the cost-effectiveness of adding tislelizumab to first-line pemetrexed-platinum chemotherapy for Chinese patients with locally advanced or metastatic nsqNSCLC without sensitizing EGFR mutations or ALK rearrangements, we estimated that first-line TPP was cost-effective regardless of the patient’s baseline characteristics.

## Data Availability

The original contributions presented in the study are included in the article/Supplementary Material; further inquiries can be directed to the corresponding author.
