# Supplementary figures and images for: The Cost-Effectiveness of Tislelizumab Plus Chemotherapy for Locally Advanced or Metastatic Nonsquamous Non-Small Cell Lung Cancer
Source: Front Pharmacol. 2022 Jul 22;13:935581. doi: 10.3389/fphar.2022.935581 (PMC9354466; doi:10.3389/fphar.2022.935581)

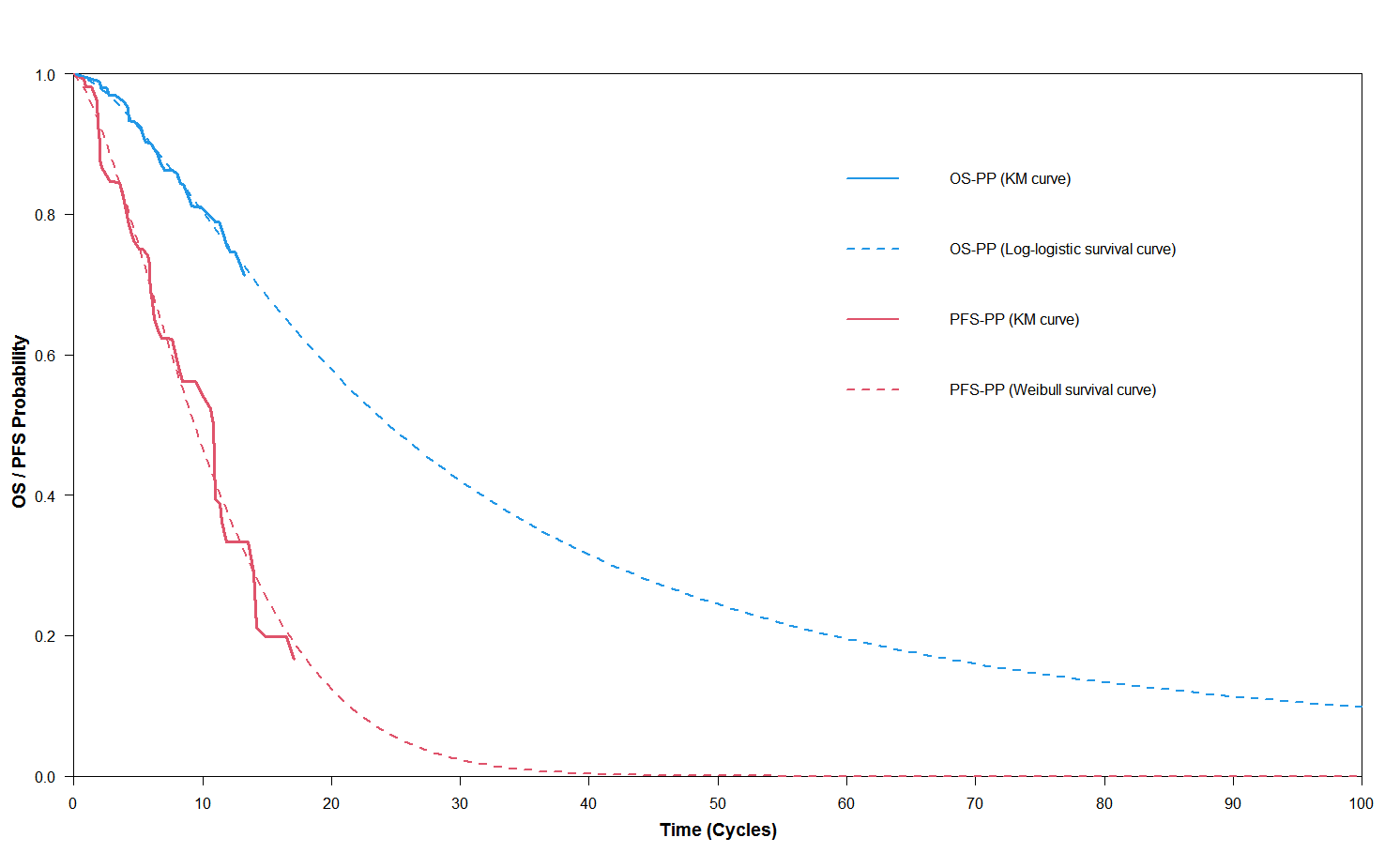

Supplement: Supplementary file 1 [file Image1.TIFF]

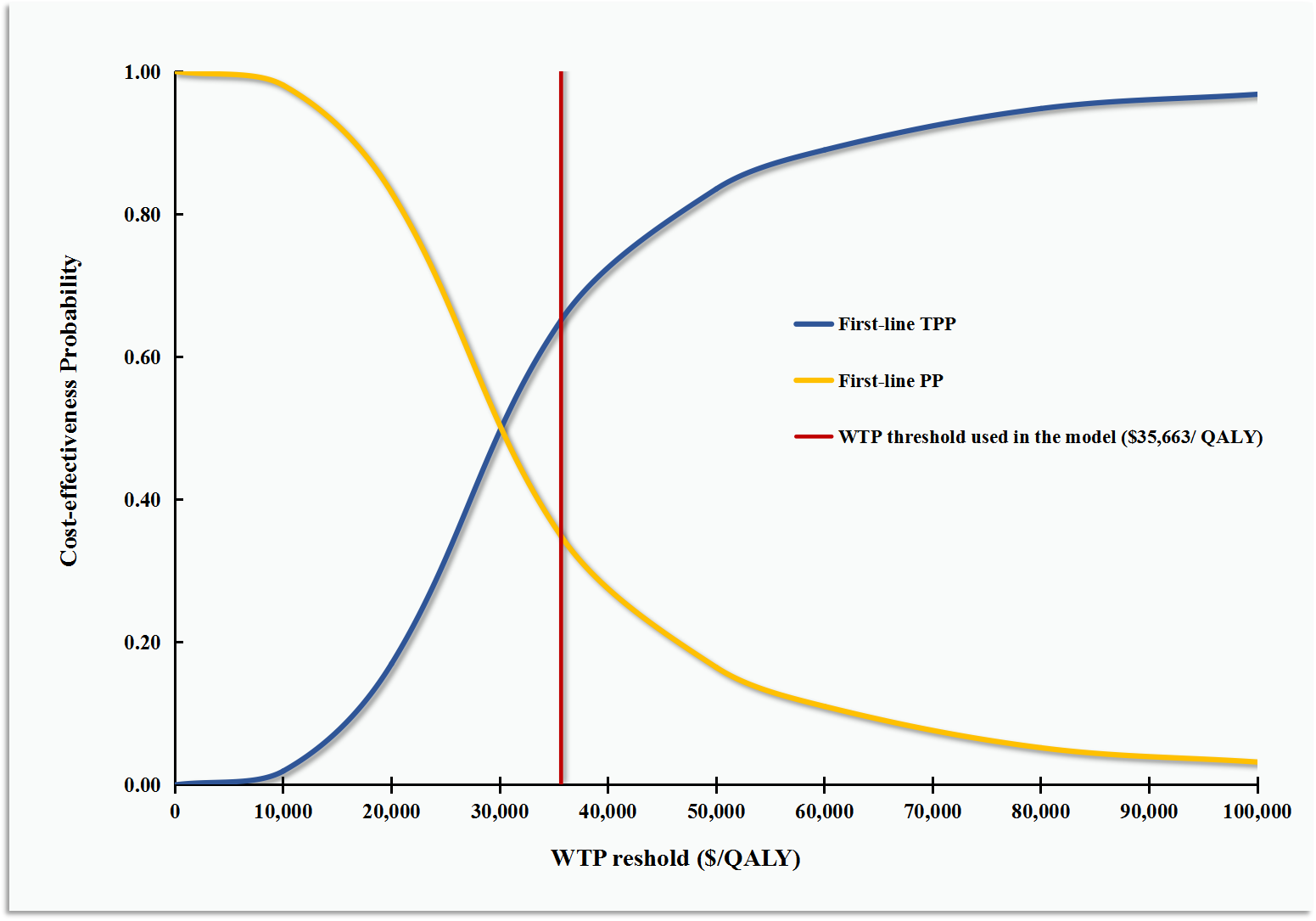

Supplement: Supplementary file 3 [file Image2.TIF]
