# Supplementary material for: The Cost-Effectiveness of Tislelizumab Plus Chemotherapy for Locally Advanced or Metastatic Nonsquamous Non-Small Cell Lung Cancer
Source: Front Pharmacol. 2022 Jul 22;13:935581. doi: 10.3389/fphar.2022.935581 (PMC9354466; doi:10.3389/fphar.2022.935581)
Supplement: Supplementary file 2 [file Table1.docx]

Table S1. Treatment dosage and administration schedule used in the model.

| **First-line treatment** | | **Premedication for the use of pemetrexed^a^** | |
| --- | --- | --- | --- |
| **Regimen** | **Dosage and Schedule** | **Drug** | **Dosage and Schedule** |
| TPP | Tislelizumab, 200 mg, consecutive cycles | Folic acid | 400 μg/d, from 5 days prior to first administration to 21 days after the last administration |
|  | Pemetrexed, 500mg/m^2^, consecutive cycles | Vitamin B12 | 1000 μg (prior to first administration),  consecutive cycles |
|  | Carboplatin, AUC 5.0 mg/ml/min or cisplatin, 75 mg/m^2^, 4-6 cycles^b^ | Dexamethasone | 24mg, consecutive cycles |
| pp | Pemetrexed, 500mg/m^2^, consecutive cycles | Folic acid | 400 μg/d, from 5 days prior to first administration to 21 days after the last administration |
|  | Carboplatin, AUC 5.0 mg/ml/min or cisplatin,75 mg/m^2^, 4-6 cycles^b^ | Vitamin B12 | 1000 μg (prior to first administration),  consecutive cycles |
|  |  | Dexamethasone | 24mg, consecutive cycles |

*TPP, tislelizumab plus pemetrexed-platinum chemotherapy; PP, pemetrexed-platinum chemotherapy; AUC,* *area under curve.*

*^a^Premedication strategy regarding the use of pemetrexed were modeled based on local clinical practice.*

*^b^Since the number of patients treated with carboplatin or cisplatin was not provided in the RATIONALE 304 trial, we assumed that they each accounted for half of the entire study patients.*
