# Supplementary material for: The Cost-Effectiveness of Tislelizumab Plus Chemotherapy for Locally Advanced or Metastatic Nonsquamous Non-Small Cell Lung Cancer
Source: Front Pharmacol. 2022 Jul 22;13:935581. doi: 10.3389/fphar.2022.935581 (PMC9354466; doi:10.3389/fphar.2022.935581)
Supplement: Supplementary file 4 [file Table2.docx]

Table S2. Parametric survival modeling of the entire patient population receiving first-line PP.

| **Parametric survival models** | **OS data** | | **PFS data** | |
| --- | --- | --- | --- | --- |
|  |  |  |  |  |
|  | **AIC** | **BIC** | **AIC** | **BIC** |
| Exponential | -217 | -213 | -117 | -113 |
| Weibull | -253 | -247 | **-174** | **-168** |
| Log-normal | -278 | -272 | -146 | -141 |
| Log-logistic | **-283** | **-277** | -152 | -147 |
| Gompertz | -247 | -240 | -172 | -165 |

*PP, pemetrexed-platinum chemotherapy; AIC, Akaike information criterion; BIC, Bayesian information criterion; OS, overall survival; PFS, progression-free survival.*
