# Supplementary material for: The Cost-Effectiveness of Tislelizumab Plus Chemotherapy for Locally Advanced or Metastatic Nonsquamous Non-Small Cell Lung Cancer
Source: Front Pharmacol. 2022 Jul 22;13:935581. doi: 10.3389/fphar.2022.935581 (PMC9354466; doi:10.3389/fphar.2022.935581)
Supplement: Supplementary file 5 [file Table5.docx]

Table S5. Model Parameters and assumptions.

| **Parameters** | **Baseline Value** | **Range** | **Distribution** | **Ref** |
| --- | --- | --- | --- | --- |
| **Survival** | | | | |
| OS of first-line PP for the entire patient population | Log-logistic θ=0.006458;κ=1.577430 | Fixed in DSA | Fixed in PSA | Estimated^a^ |
| PFS of first-line PP for the entire patient population | Weibull λ=0.02651;γ=1.45789 | Fixed in DSA | Fixed in PSA | Estimated^a^ |
| **HRs for first-line TPP vs PP** | | | | |
| OS HR in the entire patient population | 0.685 | 0.422-1.110 | Normal | Lu S, et al |
| PFS HR in the entire patient population | 0.645 | 0.462–0.902 | Normal | Lu S, et al |
| PFS HR in patients with age <65 years | 0.606 | 0.403-0.911 | Normal | Lu S, et al |
| PFS HR in patients with age ≥65 years | 0.727 | 0.407-1.297 | Normal | Lu S, et al |
| PFS HR in female patients | 0.946 | 0.487-1.840 | Normal | Lu S, et al |
| PFS HR in male patients | 0.538 | 0.367-0.789 | Normal | Lu S, et al |
| PFS HR in patients with ECOG performance status:0 | 0.834 | 0.386-1.800 | Normal | Lu S, et al |
| PFS HR in patients with ECOG performance status:1 | 0.601 | 0.416-0.868 | Normal | Lu S, et al |
| PFS HR in never smoking patients | 1.075 | 0.596-1.940 | Normal | Lu S, et al |
| PFS HR in current or former smoking patients | 0.466 | 0.311-0.697 | Normal | Lu S, et al |
| PFS HR in ⅢB nsqNSCLC patients | 0.664 | 0.319-1.379 | Normal | Lu S, et al |
| PFS HR in Ⅳ nsqNSCLC patients | 0.632 | 0.436-0.917 | Normal | Lu S, et al |
| PFS HR in patients with liver metastasis | 0.370 | 0.153-0.898 | Normal | Lu S, et al |
| PFS HR in patients without liver metastasis | 0.729 | 0.505-1.052 | Normal | Lu S, et al |
| PFS HR in patients with PD-L1 expression <1% | 0.758 | 0.469-1.224 | Normal | Lu S, et al |
| PFS HR in patients with PD-L1 expression ≥1% | 0.549 | 0.347-0.869 | Normal | Lu S, et al |
| PFS HR in patients with PD-L1 expression 1-49% | 1.058 | 0.507-2.209 | Normal | Lu S, et al |
| PFS HR in patients with PD-L1 expression ≥50% | 0.308 | 0.167-0.567 | Normal | Lu S, et al |
| PFS HR in patients with without ALK rearrangement | 0.636 | 0.434-0.930 | Normal | Lu S, et al |
| PFS HR in patients with with unknown ALK rearrangement | 0.669 | 0.345-1.297 | Normal | Lu S, et al |
| 1-Cycle probability of drug discontinuation due to AEs | | | | |
| Tislelizumab (first-line TPP) | 0.003955 | 0.001977-0.005932 | Beta | Estimated^b^ |
| Carboplatin/cisplatin (first-line TPP) | 0.004292 | 0.002146-0.006438 | Beta | Estimated^b^ |
| Pemetrexed (first-line TPP) | 0.006932 | 0.003466-0.010398 | Beta | Estimated^b^ |
| Carboplatin/cisplatin (first-line PP) | 0.001130 | 0.000565-0.001694 | Beta | Estimated^b^ |
| Pemetrexed (first-line PP) | 0.003081 | 0.001541-0.004622 | Beta | Estimated^b^ |
| Costs (US$) | | | | |
| Tislelizumab cost per 200mg | 675.84 | 337.92-1013.76 | Gamma | Local charge |
| Pemetrexed cost per 500mg/m^2^ | 281.06 | 140.53-421.59 | Gamma | Local charge |
| Carboplatin cost per 5.0 mg/ml/min | 0.40 | 0.20-0.60 | Gamma | Local charge |
| Cisplatin cost per 75 mg/m^2^ | 9.27 | 4.63-13.90 | Gamma | Local charge |
| Folic acid cost per day | 0.15 | 0.07-0.22 | Gamma | Local charge |
| Vitamin B12 cost per cycle | 0.33 | 0.16-0.49 | Gamma | Local charge |
| Dexamethasone cost per cycle | 1.14 | 0.57-1.71 | Gamma | Local charge |
| Routine follow-ups cost per cycle | 143.89 | 71.95-215.84 | Gamma | Liu Q, et al |
| Subsequent anticancer therapies cost per cycle | 854.05 | 427.03-1281.08 | Gamma | Liu Q, et al |
| BSC cost per cycle | 337.50 | 168.75–506.25 | Gamma | Liu Q, et al |
| End-of-life cost per cycle | 2627.80 | 1313.90-3941.70 | Gamma | Liu Q, et al |
| AEs management cost for first-line TPP | 1376.84 | 688.42-2065.27 | Gamma | Estimated^c^ |
| AEs management cost for first-line PP | 1031.19 | 515.59-1546.78 | Gamma | Estimated^c^ |
| Utilities | | | | |
| PFS health state | 0.856 | 0.718-0.994 | Beta | Shen Y, et al |
| PD health state | 0.768 | 0.595-0.941 | Beta | Shen Y, et al |
| AEs disutility for first-line TPP | 0.092 | 0.046-0.138 | Beta | Estimated^c^ |
| AEs disutility for first-line PP | 0.074 | 0.037-0.112 | Beta | Estimated^c^ |
| Other |  |  |  |  |
| Discount rate (%) | 5 | 0-8 | Fixed in PSA | Guidelines |
| Body surface area (m^2^) | 1.72 | 0.86-2.58 | Normal | Liu Q, et al |
| Creatinine clearance rate (ml/min) | 70 | 35-105 | Normal | Liu Q, et al |
| Proportion of patients treated with carboplatin (%) | 50 | 25-75 | Beta | Assumption |
| Proportion of patients receiving subsequent anticancer therapies (first-line TPP) (%) | 50 | 25-75 | Beta | Assumption |
| Proportion of patients receiving subsequent anticancer therapies (first-line PP) (%) | 50 | 25-75 | Beta | Assumption |

*DSA, deterministic sensitivity analyses; PSA, probabilistic sensitivity analyses; OS, overall survival; PFS, progression-free survival; TPP, tislelizumab plus pemetrexed-platinum chemotherapy; PP, pemetrexed-platinum chemotherapy; HR, hazard ratio; ECOG, Eastern Cooperative Oncology Group; NSCLC, non small cell lung cancer; PD-L1, programmed death-ligand 1; ALK, anaplastic lymphoma kinase; AEs, adverse events; BSC, best supportive care; PD, progressive disease.*

*^a^The best-fit survival distribution parameters were estimated based on the Kaplan-Meier curves from the RATIONALE 304 trial.*

*^b^Estimated in the Supplementary Table S3.*

*^c^Estimated in the Supplementary Table S4.*
