# Supplementary material for: The Cost-Effectiveness of Tislelizumab Plus Chemotherapy for Locally Advanced or Metastatic Nonsquamous Non-Small Cell Lung Cancer
Source: Front Pharmacol. 2022 Jul 22;13:935581. doi: 10.3389/fphar.2022.935581 (PMC9354466; doi:10.3389/fphar.2022.935581)
Supplement: Supplementary file 6 [file Table3.docx]

Table S3. Proportion and probability of treatment discontinuation due to AEs.

| **Treatment** | **Median OS^a^**  **(months)** | **Number of patients experiencing AEs-related drug discontinuation** | **Proportion** | **Instantaneous rate** | **1-cyle probabilities^b^** |
| --- | --- | --- | --- | --- | --- |
| First-line TPP (N=222) | 21.1 |  |  |  |  |
| Discontinuation of tislelizumab due to AEs |  | 25 | 0.112612613 | 0.003962629 | 0.003954788 |
| Discontinuation of carboplatin/cisplatin due to AEs |  | 27 | 0.121621622 | 0.004301074 | 0.004291838 |
| Discontinuation of pemetrexed due to AEs |  | 42 | 0.189189189 | 0.006955882 | 0.006931746 |
| First-line PP (N=110) | 17.1 |  |  |  |  |
| Discontinuation of carboplatin/cisplatin due to AEs |  | 3 | 0.027272727 | 0.001130202 | 0.001129564 |
| Discontinuation of pemetrexed due to AEs |  | 8 | 0.072727273 | 0.003086224 | 0.003081466 |

*AEs, adverse events; OS, overall survival; TPP, tislelizumab plus pemetrexed-platinum chemotherapy; PP, pemetrexed-platinum chemotherapy.*

*^a^The median OS for first-line TPP and PP were estimated from the best-fit parametric survival model, because these is no relevant data provided in the latest published RATIONALE 304 trial.*

*^b^The following formula was applied to convert the proportion of patient experiencing AEs-related treatment discontinuation in the clinical trial period into a 1-cylce probability:* $P=1-exp(-rt)$*, where p indicates the probability, r is the instantaneous rate and t is the time period.*
