# Supplementary material for: The Cost-Effectiveness of Tislelizumab Plus Chemotherapy for Locally Advanced or Metastatic Nonsquamous Non-Small Cell Lung Cancer
Source: Front Pharmacol. 2022 Jul 22;13:935581. doi: 10.3389/fphar.2022.935581 (PMC9354466; doi:10.3389/fphar.2022.935581)
Supplement: Supplementary file 7 [file Table4.DOCX]

Table S4. Proportion, costs and disutility of grade III/IV AEs considered in the model.

| **AEs** | **Proportion (%)** | | **Cost per event($)*^a^*** | **Disutility** |
| --- | --- | --- | --- | --- |
|  | **First-line TPP** | **First-line PP** |  |  |
| Anemia | 14.9% | 11.8% | 2150.12 | /***^c^*** |
| Leukopenia | 21.6% | 14.5% | 1267.73 | /***^c^*** |
| Thrombocytopenia | 19.4% | 13.6% | 1415.63 | /***^c^*** |
| Increased ALT | 3.6% | 2.7% | 292.59 | /***^c^*** |
| Nausea | 0.5% | 0.9% | 16.96 | 0.12 |
| Increased AST | 2.3% | 0.0% | 292.59 | /***^c^*** |
| Neutropenia | 44.6% | 35.5% | 1094.28 | 0.20 |
| Fatigue | 1.4% | 1.8% | /***^b^*** | 0.07 |
| Decreased appetite | 1.4% | 0.9% | /***^b^*** | /***^c^*** |
| Vomiting | 0.5% | 0.9% | 496.90 | 0.12 |
| Musculoskeletal pain | 0.0% | 1.8% | 7.39 | /***^c^*** |
| Rash | 0.5% | 0.0% | 26.79 | 0.10 |
| Estimated AEs costs and disutility | | |  |  |
| AEs cost for first-line TPP , $ | | | 1376.84 |  |
| AEs cost for first-line PP, $ | | | 1031.19 |  |
| AEs disutility for first-line TPP | | |  | 0.092 |
| AEs disutility for first-line PP | | |  | 0.074 |

*AEs, adverse events; TPP, tislelizumab plus pemetrexed-platinum chemotherapy; PP, pemetrexed-platinum chemotherapy; ALT, alanine aminotransferase; AST, aspartate aminotransferase .*

*^a^These AEs management costs were investigated from the local general hospitals.*

*^b^Based on the local oncologists’ opinions, these AEs do not require additional treatments.*

^c^*The disutility regarding these AEs were not reported.*
